# The Role of Body Surface Area in Quantity Discrimination in Angelfish (*Pterophyllum scalare*)

**DOI:** 10.1371/journal.pone.0083880

**Published:** 2013-12-27

**Authors:** Luis M. Gómez-Laplaza, Robert Gerlai

**Affiliations:** 1 Department of Psychology, University of Oviedo, Oviedo, Spain; 2 Department of Psychology, University of Toronto Missisauga, Mississauga, Ontario, Canada; Tulane University Medical School, United States of America

## Abstract

Although some fish species have been shown to be able to discriminate between two groups (shoals) of conspecifics differing in the number of members, most studies have not controlled for continuous variables that covary with number. Previously, using angelfish (*Pterophyllum scalare*) we started the systematic analysis of the potential influence of such continuous variables, and found that they play different roles in shoal discrimination depending on whether large (≥4 fish) or small (<4 fish) shoals were contrasted. Here, we examine the potential role of the overall body surface area of stimulus fish in shoal preference, a prominent variable not yet examined in angelfish. We report that both when numerically large (5 versus 10 fish) and when small (2 versus 3 fish) shoals were contrasted, angelfish were unable to discriminate the numerically different shoals as long as the surface area of the contrasted shoals was equated. Thus, we conclude that body surface may be an important continuous variable in shoal discrimination. This conclusion was further supported by the analysis of preference when shoals of the same numerical size but different body surface area were contrasted. We found subjects to spend significantly more time close to the shoals with the greater overall surface area. Last, we conducted an experiment in which we simultaneously controlled a set of continuous variables, including overall surface area, and found angelfish to use the number of shoal members as a cue only in large shoal contrasts but not in small shoal contrasts. This result suggests the potential existence of different processing systems for large and small numbers in fish.

## Introduction

In absence of speech, both human infants and non-human animals have been shown to exhibit a range of number-related abilities. Most often these abilities manifest as discrimination between two sets of discrete elements that differ in number. Such quantity-based judgment has been demonstrated in natural environments where in numerous species it may confer adaptive advantage in several functional contexts (e.g. foraging: [1], [2]; intergroup conflict: [3], [4], [5]; brood parasitism: [6], or reproductive decisions: [7]). Most studies on numerical competence, however, have been carried out in laboratory settings. Successful discrimination of which of two sets contains more items has been documented in preverbal infants (see [8], [9]), non-human primates [10], [11], [12] and in several other mammals, including elephants [13], [14], bears [15], dolphins [16], coyotes [17], wolves [18], and dogs [19]. Discrimination between different quantities of elements has been shown in a wide variety of other taxa, including birds [20], [21], amphibians [22], [23], fish [24], [25], and even invertebrates (reviewed in [26], [27]). Overall, the results suggest that the ability to discriminate between sets differing in the number of their elements develops during ontogenesis before the appearance of speech and also that this competence either has a common evolutionary origin (homologous functions) or a similar natural selection force (analogous functions) behind it across the variety of species studied [8].

Although many species seem to share this capacity, the question of how such relative comparisons between the sets are made has been difficult to answer. Whereas some research has suggested that infants and non-human animals are able to determine which of two sets contains more items on the basis of number alone (i.e. a true numerical process [9], [28], [29], [30], [31]), other studies have shown that non-numerical continuous variables play important roles in making the choice and the test subject could discriminate between the sets without necessarily being able to use numerical representation of the elements. For example, Clearfield and Mix [32] have shown that human infants rely on contour length or area rather than on number to discriminate small sets of items, and similar results demonstrating the use of continuous variables have been reported in other studies with human infants too [33], [34], [35]. Discrimination using continuous variables has also been reported in non-human animal species [11], [15], [16], [23], [36], [37], [38], [39].

The confusion in the literature about what aspects of the items the test subject uses in making a choice may be due to the fact that it is rather difficult to distinguish the effects of continuous variables from those of number alone as these two types of cues most often covary (see [40]). The understanding of the role potentially played by non-numerical continuous variables is an important challenge that studies on numerical competence must face.

Successful attempts have been made to prove the ability of fish to utilize numerical information in their choice. These studies attempted to control the continuous non-numerical variables and employed training procedures, i.e. relied upon learning in the studied fish species. The examples include the mosquitofish (*Gambusia holbrooki*) in which the training employed social reward [41], [42] or food reward [43], [44] but other fish species have also been utilized [24]. Another approach to study numerical competence without the potential confounding effects of non-numerical variables has been with the use of sequential presentation of items. This procedure has been employed in human infants (e.g. [45]) and non-human primates (e.g. [30]), and has also been adapted to preference tests in fish [46], [47]. In these experiments, fish continue to select the larger shoal, thus, proving their ability to rely upon numerical information alone in making shoaling decisions.

One could argue, however, that the main question is not whether an experimenter could force fish to use numerical information alone in artificial laboratory procedures, but rather what the natural, spontaneous (untrained) behaviour of the studied fish species may be. That is, when presented with a task that may be solved in multiple ways the question is what strategy the fish would choose. The spontaneous binary shoal choice task may allow us to answer this question. In this task the test fish is presented with a shoal on each of the opposite sides of the test tank and can choose between the two shoals of conspecifics that differ in the numerical size of their members. Choice is quantified as the relative distance to one vs. the other side of the tank. The live stimulus fish move, change location and orientation, modify their inter-individual distance, move into overlapping positions, i.e., dynamically alter numerous continuous variables including the total visible surface area, the linear dimensions and the density of the shoal.

This binary choice paradigm has been the most frequently employed method in studies of numerical competence. In a number of fish species, results show that subjects spontaneously discriminate and prefer the larger of the two shoals (swordtails [48]; mosquitofish [49]; guppies [50]; zebrafish [51]; angelfish [52], [53]; red tail splitfin [25]). Many fish when placed alone in a novel, potentially dangerous, environment (the test tank) seek protection in larger groups, an effective antipredatory strategy [54]. The response, thus, appears to have an adaptive value and fish exhibit this behaviour spontaneously, i.e. in the absence of prior training.

We have recently started to isolate the potentially operative continuous non-numerical variables and systematically analyzed the role played by the most prominent of them, one at a time, to understand their influence on quantity discrimination. Our previous research using the binary shoal choice paradigm, has consistently shown that the individual angelfish (*Pterophyllum scalare*) prefers to spend more time close to the larger of two groups of conspecifics (shoals) placed in opposite sides of the test tank. This choice was observed both when using large shoals (≥4 members [52]), e.g. for comparisons between 5 vs. 10 fish [55], and when using small ones (<4 members), e.g. 2 vs. 3 fish shoals [53]. We found that several continuous variables, including shoal density, linear extent, or inter-fish distance, individually considered [53], [55], as well as the overall swimming activity of the stimulus shoals [56] had diverse effects on performance of the test fish. Some of these continuous variables were indeed found to affect the choice between numerically different shoals made by angelfish, but the relevance of these continuous variables was also found to depend upon the numerical size of the contrasted shoals.

We have not studied the potential effect of an important non-numerical continuous variable, the surface area of the contrasted shoals. As the surface area encompassed by the larger shoal is greater than that of the smaller one, this variable could, in principle, be used by angelfish to judge the size of a shoal. The goal of the current study is to examine the role of this continuous variable. First, we sought to replicate and confirm some of our previous findings and used two contrasts, shoals of large number of individuals (5 vs. 10 fish) and shoals of small number of individuals (2 vs. 3 fish), tests in which all cues, continuous and numerical, were available (baseline performance). Subsequently, also using the above large and small shoal contrasts, we attempted to control for body surface area of the stimulus fish by minimizing the difference in total surface area of the contrasted shoals. We investigated whether angelfish could still distinguish between the larger and the smaller shoals under these circumstances. In addition, we also performed the opposite manipulation, i.e. in which angelfish were exposed to pairs of shoals of equal numerical size (5 vs. 5 fish, and 3 vs. 3 fish), but differing in body surface area. A preference for the shoal with the greater overall surface area in this treatment would underscore the importance of this variable. Finally, we conducted an experiment in which several continuous variables, including surface area, were simultaneously controlled and thus we asked whether angelfish could discriminate numerically different shoals (5 vs. 10 fish, and 2 vs. 3 fish) without these continuous variables playing any potential role.

## Materials and Methods

### Ethics Statement

The experiments described here comply with the current laws of the country (Spain) in which they were performed and were approved by the Committee on the Ethics of Animal Experiments of the University of Oviedo (permit number: 13-INV-2010).

### Subjects and holding conditions

**Figure 1:**
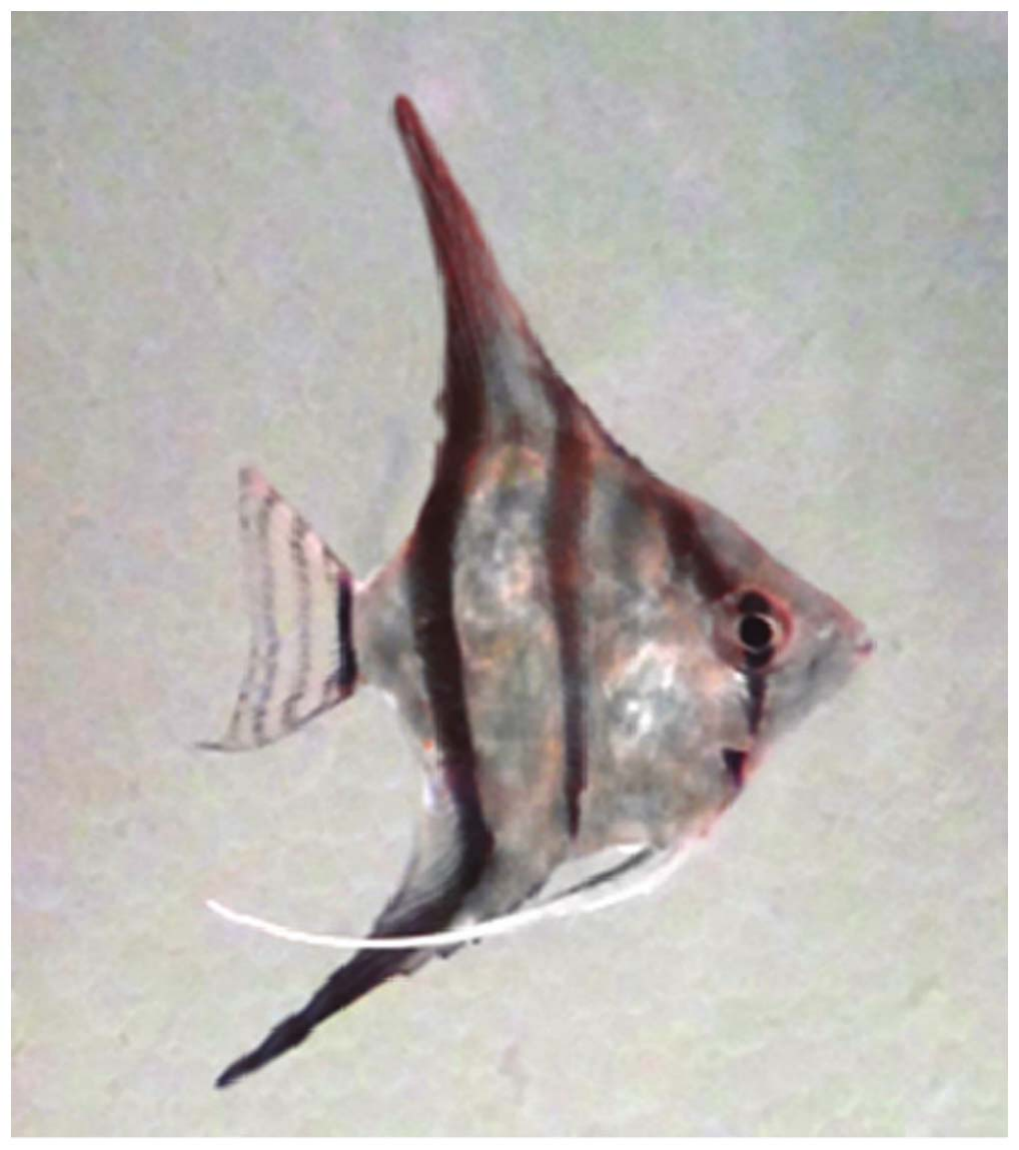
One of the experimental angelfish (*Pterophyllum scalare*).

Wild type juvenile angelfish (*Pterophyllum scalare*) were obtained from local commercial suppliers (Fig. 1). Only juveniles of this sexually monomorphic species were studied so as to eliminate possible confounding effects arising from courtship or agonistic/territorial interactions. The fish were housed in glass holding aquaria (length × width × depth: 60 cm×30 cm×40 cm) in groups of 20–22 when fish were of small and medium size, and in groups of 15 for large size fish. All fish were allowed a minimum of a two-week acclimation period before behavioural testing.

Test fish and stimulus fish (which were used to elicit test fish behaviour) were randomly chosen from the same cohort and were housed separately, with no visual and olfactory communication being possible between fish in the separate aquaria. Fish of different size were also housed separately. Aquaria were filled with dechlorinated tap water. Temperature of the water was kept at 25°C using thermostat-controlled heaters. Each aquarium was illuminated by a 15-W white fluorescent light tube placed above the tank. A 12∶12-h light:dark cycle was maintained with lights on at 08.30 hour. External filters continuously cleaned the aquaria, which had a 2-cm deep gravel substratum. The fish were fed commercial fish food (JBL GALA, JBL GmbH & Co. KG, Neuhofen, Germany) twice daily, at 10.00 h and at 18.00 h.

### Apparatus

**Figure 2:**
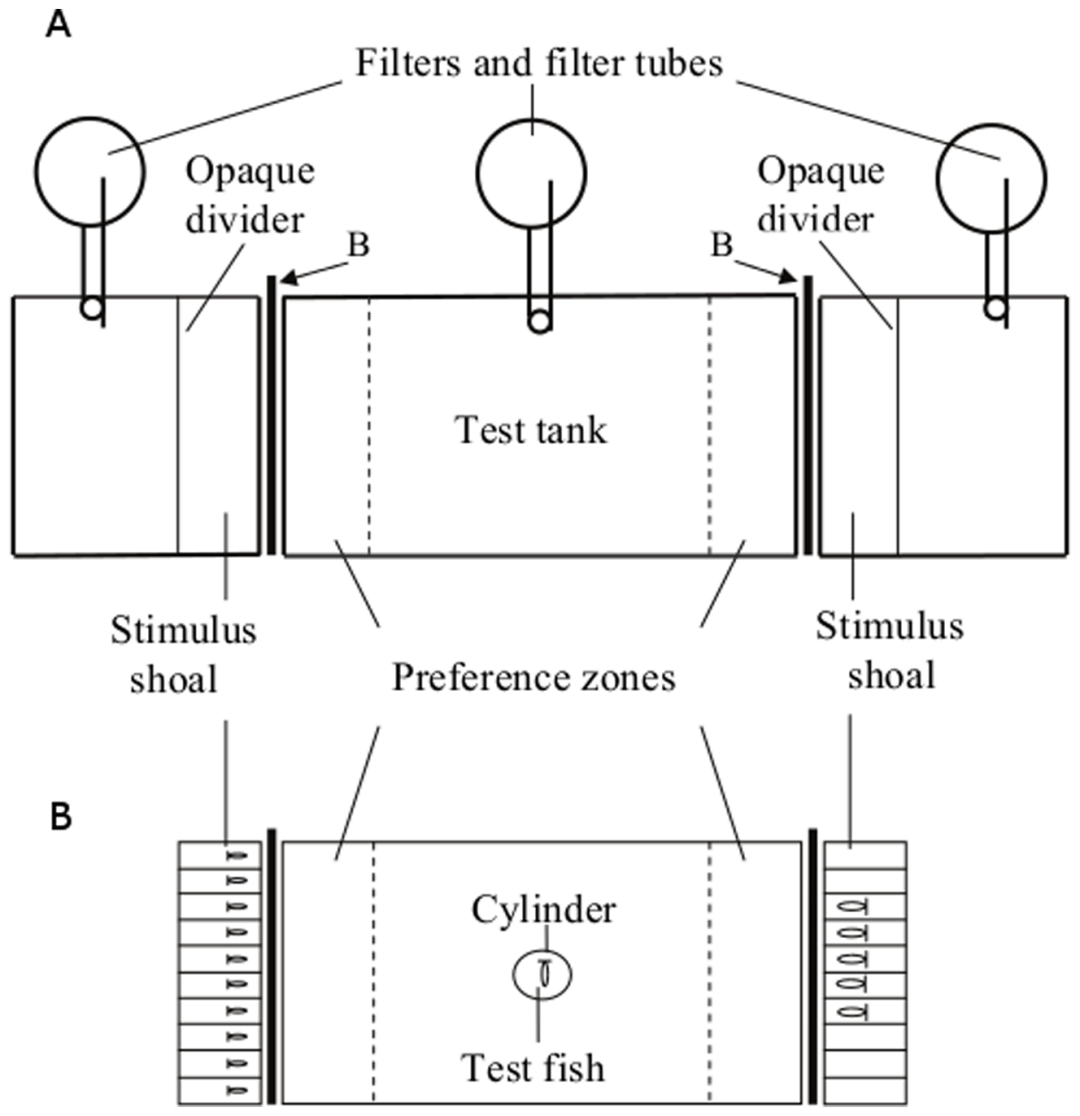
The experimental apparatus. (A) Diagram of the experimental apparatus showing the central test tank and the two stimulus tanks at each end of the test tank. Removable opaque white dividers were used to separate a 10-cm compartment close to the test tank, where the stimulus shoals were presented to the test fish. Opaque white barriers (B) were used to visually isolate the two stimulus tanks (containing the stimulus shoals) from the test tank. These barriers were removed when preference tests commenced. The time test fish spent within 10 cm of the stimulus shoals (preference zones) was recorded. (B) The test tank and the stimulus compartments. Diagram showing the test tank and the two stimulus compartments at each side. To simultaneously control for several continuous variables, the stimulus compartments were divided into 10 identical sectors by transparent Plexiglas partitions and each fish of the stimulus shoals was individually placed into each of the adjacent sectors. An example of 5 vs. 10 fish contrast is shown (Exp. 1). When shoals of 2 vs. 3 fish were contrasted (Exp. 2), fish were placed into the adjacent central sectors.

The experimental apparatus to assess spontaneous shoaling preference in binary choice tests was similar to what we used in a previous study [56]. It consisted of a test aquarium with one stimulus aquarium positioned at each end (Fig. 2a). The test aquarium was identical in all respects to the holding aquaria and was maintained under the same conditions. The stimulus aquaria were of smaller dimensions (30×30×40 cm depth) but the side facing the test aquarium was of the same size as the short lateral sides of the latter (30×40 cm). Other conditions (e.g. water quality and temperature) were identical to those of the holding and test aquaria. A divider isolated a 10-cm compartment in the stimulus aquaria where the stimulus shoals were presented. Before preference tests commenced, the stimulus shoals were placed in the part of the stimulus aquaria outside of the stimulus compartment. Except for the front, all exterior walls of the aquaria that were not adjacent to other aquarium walls were lined with white cardboard to prevent the fish from being influenced by external visual stimuli. Removable opaque white barriers placed outside the two end sides of the test aquarium were used to visually isolate the latter from the stimulus aquaria and these barriers were removed when preference tests commenced (B in Fig. 2a).

Five vertical lines drawn on the front and back walls of the test aquarium at a distance of 10 cm divided the test aquarium into six equal zones and facilitated measurements of the test fish's movements and position. The two 10-cm zones closest to the stimulus aquaria were considered to be the preference zones. At least three-quarters of the body length of the fish had to be within the boundary for the fish to be included in a particular zone. Swimming activity of test fish was measured as the frequency (number of times) the fish crossed the lines drawn on the walls of the aquarium during the tests.

### General procedure: Preference tests

The experimental procedure was the same as that adopted in a previous study [55]. Briefly, in each trial a single test angelfish was given a choice between two numerically different shoals of conspecifics presented simultaneously and positioned in the stimulus aquaria on opposite sides of the test aquarium. The chosen number of fish that served as stimulus shoals were taken at random from the stimulus fish holding aquaria and were gently placed into the part of the stimulus aquaria outside of the stimulus compartment. To control for any potential side bias the allocation of the shoals to the stimulus aquaria was initially determined at random and then counterbalanced across trials. All fish were allowed a 15-min acclimation period in the new aquaria (see below). Trials took place 15–30 min after feeding in the morning.

Test fish were randomly selected from a test fish holding tank, and were introduced singly to the centre of the test aquarium. Test fish were allowed to swim freely with the barriers between aquaria removed, so they could see the 10-cm compartments where the stimulus shoals would be presented. This acclimation period in the absence of stimulus shoals lasted for 15 min and also allowed stimulus shoals to settle in the respective stimulus aquaria. At the end of this period, the barriers between aquaria were replaced and the stimulus shoals were gently shepherded to the 10-cm stimulus compartment. The test fish was confined in the centre of the test aquarium via a transparent, open-ended, plastic cylinder (7 cm diameter), in which it remained for 2 min. During this time, the opaque white barriers between the aquaria were removed to reveal the stimulus shoals, thus allowing the confined test fish to view the stimulus shoals at both sides of the test aquarium from an equal distance. The start cylinder was then gently raised and the test fish released. Shoaling behaviour, recorded over a 15-min period, was defined as the time spent by the test fish in the 10-cm preference zones, i.e., within 10 cm from the wall adjacent to the stimulus shoal aquaria on either side. Behavioural responses of the test fish were recorded with a video camera (Sony video Hi8, model CCD-TR750E) concealed behind a blind. The recordings were later replayed for analysis.

At the conclusion of the recording session, the barriers between aquaria were replaced and the positions of the stimulus shoals were interchanged between stimulus aquaria to control for any potential directional bias. After a second 15-min settling interval, another 15-min observation period was run with the same test fish following the same procedure as described above. After the second observation period, the aquaria were cleaned before being replenished with dechlorinated tap water. None of the fish in the stimulus shoals were used as test fish and vice versa. Within each experiment, the order of testing was randomized according to different treatment conditions. Stimulus shoals were rearranged after each session, so that each test fish was exposed to a different stimulus fish set. The fish were returned to the suppliers at the end of the study.

### Experiment 1: Discrimination of large shoals (5 vs. 10) and control for surface area of the stimulus fish

In this experiment, we first attempted to replicate our previous finding [55] that demonstrated the angelfish's ability to discriminate between large shoals when the ratio was 1∶2. Test fish of similar body size to that of the stimulus fish were presented with a binary choice between a shoal of five conspecifics versus a shoal of 10 conspecifics. The number of test fish tested in this task was 12.

To examine whether the choice made by the test fish was based upon, or was influenced by, the overall body surface of the shoal members rather than their number, we measured the performance of a new sample of 12 naïve fish. Now, we controlled for the body surface area by minimizing the difference in this continuous variable between the stimulus shoals. To achieve our goal we first took photographs of the fish with a digital camera and using the tpsDig software [57] we calculated both the body surface area (excluding fins) and standard length of stimulus fish from the digitized images. To allow for accurate calibration of photographs a background of 1 mm graph paper was placed behind the fish. For surface area, 16 points (‘landmarks’) were defined on each individual in tpsDig. Fish larger than the subjects were selected to constitute the smaller stimulus shoal (that of five fish) whereas fish smaller than the subjects were selected to constitute the larger stimulus shoal (that of 10 fish), in such a way that the overall body surface of the five large fish was approximately equal to that of the 10 small fish (body surface areas and standard lengths of the stimulus fish and test fish in all the treatments are summarized in Table 1).

**Table 1:** Body surface area and length of the fish.

	Experiment 1 and 2		Experiment 1		Experiment 2	
Measurements	Test fish [32]	Stimulus fish Intermediate [40]	Stimulus fish Large fish [40]	Stimulus fish Small fish [40]	Stimulus fish Large fish [40]	Stimulus fish Small fish [40]
Body surface area (mean ± S.E.M.)	3.66±0.04	3.63±0.04	4.82±0.13	2.43±0.07	4.58±0.07	2.99±0.06
Standard length (mean ± S.E.M.)	3.02±.04	2.97±0.05	3.55±0.05	2.03±0.05	3.44±0.04	2.39±0.04

Body surface area (cm^2^) and standard length (cm) of the test fish and stimulus fish used in experiments and contrasts. The sample size taken for measurement of each fish size is indicated in square brackets [ ].

In the subsequent treatment, we kept the number of fish in the contrasted shoals identical but we made the surface area of these shoals different. Both shoals were composed of five fish (5 vs. 5 fish) but using the above described digital photography analysis and selection procedure, one shoal was made to consist of individuals with larger overall body surface area than the test fish and the other to have individuals with smaller body surface area than the test fish's. The result of this arrangement was that the total surface area of one stimulus shoal was nearly double that of the other stimulus shoal. Another 12 naïve test fish were studied in this test.

Finally, in an attempt to simultaneously control for a set of continuous variables we minimized differences between stimulus shoals (5 vs. 10 fish) in overall surface area, swimming activity, density and inter-fish distance hoping that numerical information alone would be the only prominent difference between the contrasted shoals. We employed two removable transparent Plexiglas frames with 10 individual small identical sectors that were introduced into each stimulus compartment (see Fig. 2b). Stimulus shoals of similar overall body surface area were confined in these small sectors, thus providing control over the above mentioned variables [56]. A set of 12 naïve test fish were used.

### Experiment 2: Discrimination of small shoals (2 vs. 3) and control for surface area of the stimulus fish

Previous studies indicated the existence of a ‘set size limit’ of three fish for small numbers in angelfish [53] and suggested that this species uses a different mechanism of discriminating these small shoals as compared to how they distinguish large (more numerous) shoals. The present experiment entailed identical protocols to those described in Experiment 1, except the test fish were given a choice between two small stimulus shoals, one composed of two conspecifics and the other composed of three conspecifics. As above, the experiment had four treatments: one, replication of prior analysis of discrimination between shoals of 2 vs. 3 fish (baseline, all continuous and numerical information available to test fish); two, a treatment in which the total body surface area of the stimulus shoals contrasted was minimized; three, contrasted shoals with same number of shoal members (3 vs. 3 fish) but with different overall body surface area (one shoal had approximately one and a half larger overall body surface area than the other); and four, a treatment in which overall surface, swimming activity, density and inter-fish distance were all simultaneously controlled but the shoals differed in numerical size (2 vs. 3 fish). As in Experiment 1, a naïve set of 12 test fish was studied in each of the four treatments of Experiment 2.

### Statistical analysis

The time spent in the preference zones was recorded as a measure of each test fish's preference for a particular stimulus. We calculated a preference index for each test fish as follows: time spent in the preference zone near the larger stimulus shoal was divided by the total time spent shoaling (i.e., the time spent within 10 cm from either stimulus shoals). A preference index equalling 1 would indicate complete preference for the larger shoal, whereas an index value of 0 would indicate complete preference for the smaller shoal. In the treatments with equal number of fish in the contrasted shoals, the preference index was calculated similarly but the numerator referred to the shoal with greater overall surface area. A one sample two-tailed t–test was used to compare the observed proportions against a chance value of 0.5 (null hypothesis). The proportions were normally distributed. Statistical probabilities reported are two-tailed. The null hypothesis was rejected when its probability (*P*) was less than 0.05.

The effect of the treatments on preference was investigated with one-way ANOVA for independent samples. In case of a significant effect, Tukey Honestly Significant Difference (HSD) post hoc multiple comparison test was performed to determine which treatment group significantly (p<0.05) differs from one another.

## Results

### Experiment 1: Discrimination of large stimulus shoals and control for surface area

In all trials, test fish visited both preference zones and thus had the opportunity to assess each stimulus shoal. Overall, the level of swimming activity (number of lines crossed) shown by test fish prior to stimulus presentation was significantly higher than that exhibited when in the presence of the stimulus shoals (mean ± SEM: 67.67±5.24 and 50.87±3.92, respectively; paired *t-test*: *t*_47_ = 4.854, *P*<0.001). The reduced shuttling activity during the presence of stimulus shoals is due to subjects staying longer in the preference zones close to the stimulus fish. This activity pattern was confirmed in all treatments (*P*s≤0.043), except in the treatment in which differences in overall surface area of fish were reduced between the stimulus shoals (mean ± SEM: 56.50±9.62 and 45.29±6.11, respectively; *t*_11_ = 2.057, *P* = 0.064), suggesting a greater difficulty in decision making subjects moving more frequently from one stimulus shoal to the other.

**Figure 3:**
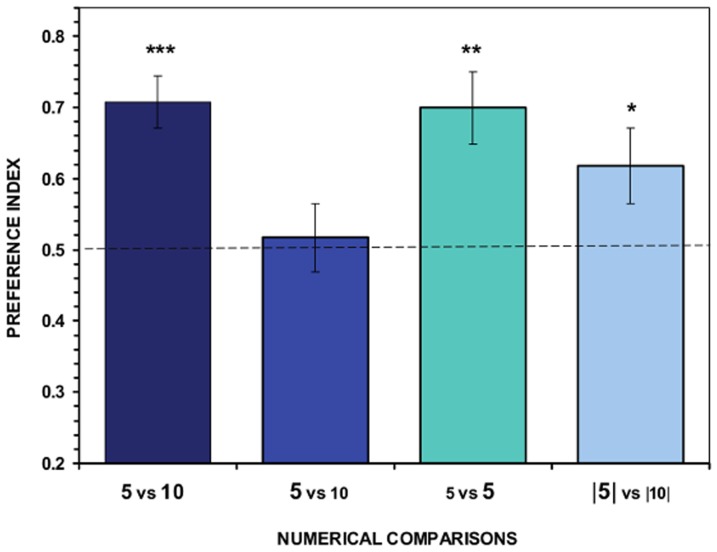
Results of Experiment 1. Proportion of time (*preference index*, Mean ± SEM) test fish spent in close proximity to the stimulus shoals. Values above 0.5 indicate a preference for the more numerous shoal of stimulus fish or a preference for the stimulus shoal with the greater overall surface area when the stimulus shoal is of the same numerical size. In the X-axis legend the numbers indicate the number of members of the contrasted shoals. We also illustrate the size of the body surface area: the large font size means that the body surface area of the stimulus fish was large and the smaller font size indicates that the surface area was small. Note that in case of the second condition (second bar) the total surface area of the two contrasted shoals was made similar by increasing the surface area of the individual stimulus fish in the less numerous shoal and decreasing it in the more numerous shoal. Numbers in between bars **|** **|** (fourth condition) represent stimulus shoals confined in small sectors of a transparent compartment which was designed to minimize differences in several continuous variables that may have covaried with the sizes of these shoals (see text for details). Significant departure from the null hypothesis of no shoal preference is indicated by asterisks: * *P*<0.05; ** *P*<0.01; *** *P*<0.001.

The initial treatment, in which stimulus fish of similar size were presented to the test fish (i.e. no control for surface area), confirmed that angelfish are able to discriminate between large stimulus shoals of conspecifics that differ in a 1∶2 ratio (5 vs. 10 fish). Fish spent significantly more time near the larger shoal over the smaller one (*t*_11_ = 5.728, *P*<0.001; Fig. 3). When the body surface area of the fish in the stimulus shoals was controlled by minimizing the difference in total surface area of the contrasted shoals (5 vs. 10), the fish did not exhibit any significant preference for either of the shoals, i.e. the test fish performed at chance (*t*_11_ = 0.360, *P* = 0.725; Fig. 3). This result indicates that fish are sensitive to the overall body surface area of the stimulus shoals and that this variable affects discrimination of quantities in angelfish. The effect of the body surface area of the stimulus fish was further supported when we investigated the potential role of this variable per se. Two shoals of the same numerical size (5 vs. 5) but differing in surface area, were presented, and the test subjects showed significant preference for, i.e. stayed closer to the shoal with the greater total body surface area (*t*_11_ = 3.863, *P* = 0.003; Fig. 3). Finally, when we minimized the potential differences in several continuous variables simultaneously (including body surface area), test subjects showed a significant preference for shoaling with the larger, i.e. more numerous shoal (10 fish) versus the smaller (less numerous) shoal (5 fish) (*t*_11_ = 2.242, *P* = 0.047; Fig. 3, right most bar).

One-way ANOVA showed a significant difference between the magnitude of the preferences among the four treatment groups (*F*_3,44_ = 3.504, *P* = 0.023), and the Tukey HSD test indicated that the group of fish receiving the equated overall surface area of the stimulus shoals was significantly different from that for which the total body surface area was not equated between the contrasted shoals (*P* = 0.033) and from the treatment group that received the same numerical size shoals (*P* = 0.044). These results support the notion that the body surface area of the shoals plays a role in shoal discrimination in angelfish. No significant difference was found between the magnitude of the preference between the group for which the treatment included controlling for the overall surface area of the shoals and the treatment controlling for several non-numerical variables (*P* = 0.439). The performance in the latter treatment group was also non-significantly different from performance of any of the other treatment groups. The differences (or lack thereof) found among the treatment groups in preference cannot be attributed to time spent by the test fish shoaling near the stimuli during the tests (One-way ANOVA: *F*_3,44_ = 1.829, *P* = 0.156).

### Experiment 2: Discrimination of small stimulus shoals and control for surface area

The results we obtained using small stimulus shoals were similar to those found with large shoals. All test fish entered both preference zones of the test aquarium in the presence of the stimulus shoals. Overall, during the acclimation period with no stimulus shoals, subjects also exhibited a significantly higher swimming activity as compared to that shown in the presence of the stimulus shoals (mean ± SEM: 65.38±4.72 and 40.92±3.25, respectively; paired *t-test*: *t*_47_ = 4.011, *P*<0.001). This pattern was not maintained in the treatments in which total surface area of the contrasting stimulus shoals was minimized (mean ± SEM: 60.33±7.28 and 38.08±9.20, respectively; *t*_11_ = 1.775, *P* = 0.104) and when diverse continuous variables were simultaneously controlled (mean ± SEM: 69.08±13.15 and 41.17±6.97, respectively; *t*_11_ = 1.708, *P* = 0.116). In these latter treatments fish did not significantly reduced shuttling activity relative to the acclimation period, which coincided with no clear preference exhibited by the test fish for either shoal.

**Figure 4:**
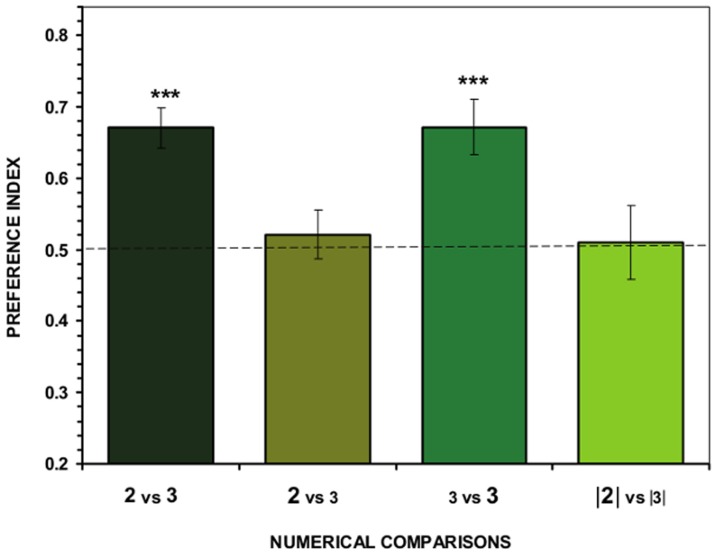
Results of Experiment 2. Proportion of time (*preference index*, Mean ± SEM) test fish spent in close proximity to the stimulus shoals. Values above 0.5 indicate a preference for the more numerous shoal of stimulus fish or a preference for the stimulus shoal with the greater overall surface area when the stimulus shoal is of the same numerical size. In the X-axis legend the numbers indicate the number of members of the contrasted shoals. We also illustrate the size of the body surface area: the large font size means that the body surface area of the stimulus fish was large and the smaller font size indicates that the surface area was small. Note that in case of the second condition (second bar) the total surface area of the two contrasted shoals was made similar by increasing the surface area of the individual stimulus fish in the less numerous shoal and decreasing it in the more numerous shoal. Numbers in between bars **|** **|** (fourth condition) represent stimulus shoals confined in small sectors of a transparent compartment which was designed to minimize differences in several continuous variables that may have covaried with the sizes of these shoals (see text for details). Significant departure from the null hypothesis of no shoal preference is indicated by asterisks: *** *P*<0.001. Note that the difference between figures 3 and 4 is that in the latter the number of fish in the contrasted shoals is small (2 vs. 3).

Subjects significantly preferred the larger shoal (3 fish) to the smaller one (2 fish) when individual fish within the shoals had similar body surface area (*t*_11_ = 5.970, *P*<0.001; Fig. 4). However, as in Experiment 1, subjects failed to discriminate between the two shoals (2 vs. 3) when the overall body surface area was similar in both shoals (*t*_11_ = 0.623, *P* = 0.546; Fig. 4). This result suggests that the choice of angelfish is influenced by total surface area of the stimulus shoals both when numerically large (previous experiment) and when small (current experiment) stimulus shoals are contrasted. Consistent with the latter finding, a significant preference for the shoal with the greater overall body surface area was found when the stimulus shoals had the same number of fish (3 vs. 3: *t*_11_ = 4.479, *P* = 0.001; Fig. 4). However, the test subjects could not distinguish between shoals of 2 vs. 3 fish, and did not perform significantly differently from chance, when several continuous variables were simultaneously controlled (*t*_11_ = 0.202, *P* = 0.843; Fig. 4, right most bar).

A subsequent one-way ANOVA showed a significant difference between the magnitude of the preferences of the four conditions (*F*_3,44_ = 5.255, *P* = 0.003), and the Tukey HSD test indicated that test fish for which the overall surface area of the stimulus shoals was equated performed significantly differently from, i.e. lower than, test fish in the treatment group for which the total surface area of the contrasted shoals was not minimized (*P* = 0.047) and also compared to test fish whose choice was between shoals of the same numerical size (*P* = 0.045), again confirming that discrimination of shoals was affected by the total surface area of the shoals. Performance of the test fish under the latter two conditions also differed significantly from performance of fish in the condition in which several variables (including surface area) were equated for the contrasted stimulus shoals (*P*s≤0.029). These differences in shoaling preference were not due to putative differences in total time spent by the groups in shoaling behavior. One-way ANOVA showed no significant difference between treatment groups in this parameter (*F*_3,44_ = 2.055, *P* = 0.120).

## Discussion

Previously it has been shown that numerous species, including the angelfish, can discriminate between large sets of elements as long as the ratio between the sets reaches 1∶2 ratio and between small sets composed of 2 vs. 3 elements. Our current results confirm these previous findings and show that angelfish reliably prefer the larger shoal (1∶2 ratio for large sets, i.e. 5 vs. 10 fish; and 2 vs. 3 fish for small sets). However, performance in these studies could be influenced by both numerical characteristics of the contrasted sets and the continuous variables that covary with number.

We have started the investigation of whether and which continuous variable(s) may influence or guide angelfish when making a choice between shoals of conspecifics differing in numerical size. In the current study, we focussed on a previously uninvestigated continuous variable, the body surface area of the stimulus fish. We found overall surface area of the shoal to have a strong influence on the shoal choice (larger surface area shoals were preferred). Comparable results indicating that body surface area of the stimulus fish plays an important role in the discrimination were found both when numerically large shoals (Experiment 1, 5 vs. 10 fish) and also when numerically small shoals (Experiment 2, 2 vs. 3 fish) were contrasted. We arrived at this conclusion on the basis of two separate sets of findings. One, when we minimized the difference between the contrasted shoals in their overall body surface area, despite numerical differences between the shoals, no significant preference was shown by angelfish towards either shoal. Two, when we used shoals of identical number of conspecifics differing in the total body surface area, angelfish consistently chose the shoal with the larger surface area.

Mosquitofish were also shown to rely on this continuous variable: when the contrasted shoals presented to this species had similar surface areas, mosquitofish chose randomly both when numerically large or when numerically small shoals were contrasted [49]. Likewise, mosquitofish trained to discriminate sets of geometric figures failed the discrimination of the trained stimuli when the cumulative surface area of the geometric figures was matched [41], [42]. Very young guppies also failed the trained discrimination between two small sets of dots when the area of the contrasted dots was equal [58], although they did discriminate between large sets under these circumstances [44]. Surface area appears to be an important non-numerical variable that has also been demonstrated to provide a basis for discrimination between quantities in other animal species and in different contexts [15], [16], [34], [39].

In studies dealing with size assortment in shoals, it has been shown that fish are capable of discriminating between conspecifics of different size using only visual cues, even when the size differences are smaller than in the current study [59], [60], [61]. A preference for shoaling with large conspecifis over smaller ones has been shown in a number of fish species such as European minnows [61], two-spotted gobies [60], guppies [62] and mosquitofish [63], [64]. Although this behaviour has associated costs, such as increased resource competition, the preference may indicate that benefits (e.g. finding food faster) are greater than the costs. On the contrary, reluctance by large fish to join small ones may reflect a greater cost [61]. The preference is often thought to be driven by predation risk and it has been suggested that the oddity effect is likely to prevent larger fish from joining shoals of smaller individuals [65]. In our study we could not distinguish whether our test fish preferred the overall surface area of the shoal or the surface area of its individual members.

Despite the above uncertainty, our results clearly show that body surface area is an important factor, a continuous variable upon which angelfish can make its decision about which shoal to choose. This result taken together with our prior findings that suggested the role of other non-numerical variables suggests that angelfish may indeed use a variety of cues when discriminating between shoals of conspecifics. However, the question remained: can angelfish base their shoaling decision upon numerical information alone? Previously, our results implied that they can, but only when the task involved distinguishing between numerically small sets. In this latter case, angelfish hardly relied upon continuous variables [53], [55], [56]. In the current study, we attempted to more directly answer this question by minimizing the difference between the contrasted numerically different shoals in a number of possibly important continuous variables. Under these circumstances, we found angelfish to still be able to show a significant preference for the numerically larger shoal, but only when the contrasted shoals had large number of members (5 vs. 10). Our results indicate that angelfish may not have to rely on inter-fish distance, density, swimming activity or body surface area for estimation of shoal size but instead may be able to utilize numerical cues. This conclusion, however, contradicts our findings showing that when the contrasted shoals were equated with regard to total body surface area no significant preference was exhibited by the test fish. Although in our last treatment we made every attempt to control all non-numerical variables, it is possible that we missed some. It is possible, for example, that our experimental manipulations while addressing the intended continuous variables made certain features of stimulus shoals, other continuous variables, more salient. The linear extent occupied by the stimulus shoals may be one such variable: large shoals occupy longer extent. Notably, however, in a previous work, when linear extent of the shoals was individually controlled, this variable was found not to have a significant influence on the discrimination [55]. These controversies highlight an important problem: when using living animals as stimuli the simultaneous control of all continuous variables may not be entirely possible [40], a problem to which we return below.

Numerical information driving the selection of the larger quantity, both with large and small numbers, has been claimed in a number of fish species when non-numerical variables were controlled for by using a paradigm involving spontaneous discrimination of sequential presentation of fish in the stimulus shoals [46], [47], and also by training fish to discriminate between patterns of geometric figures [41], [42], [66]. Our results now clearly demonstrate that angelfish are able to attend both to continuous variables and to numerical information, and perhaps they may be able to utilize these two types of information simultaneously. It is also plausible that even in nature, depending on the context or the particular characteristics of the situation, the relative salience of these features may be different and angelfish use them accordingly. Such context specificity has been shown in other species too. For example, human infants and non-human primates attend to number over continuous variables when tested with large sets of objects [9], [28], [30].

The results of Experiment 2 with small number of fish in the contrasted shoals showed that our test fish did not discriminate the numerically larger shoal when non-numerical variables were controlled and equated between the shoals. This result is in apparent contradiction to what we found in prior studies in which we obtained little evidence for the use of non-numerical variables in contrasts between small shoals. It is notable, however, that our procedure used live stimulus fish, and it is plausible that live fish can provide numerous cues other than those we controlled and were aware of. For example, stimulus fish may provide subtle behavioural cues and a smaller number of stimulus fish confined and controlled in a manner we did in Experiment 2 may behave differently from the large number of stimulus fish we employed in Experiment 1. Such difference could have influenced the response of our test fish, a working hypothesis that we will test by conducting a detailed behavioural analysis of our stimulus fish. Furthermore, it is also notable that lack of preference for the more numerous shoal when the contrasted shoals had only a small number of fish in them may be due to the ‘oddity effect’ [67]. Fish that appear different from their shoal mates may stand out and may be easily detectable for predators. Thus fish tend to prefer shoals with members whose characteristics (size, colour) are similar to their own. In case of a pair of shoals of two large fish vs. three small fish the test fish may be facing a conundrum: the more numerous shoal has only three individuals and thus it will not provide much more protection but all three are smaller and thus the joining test fish may stand out as the largest target (and thus potentially more attractive to predators). The smaller shoal (two fish) may provide even less protection but at least the joining test fish will be the smallest in the group. Such possible balancing aspects of body size and shoal member number may not happen the same way in more numerous shoals, where the effect of the larger number of members may dominate (the test subject may not stand out that much but the larger number of shoal members may provide better antipredatory protection). Whether the above speculation is correct will be experimentally tested in the future.

Nevertheless, using a training procedure to discriminate between patterns of geometric figures, Miletto Petrazzini et al. [58] found similar results to ours in newborn guppies. These fish were successful in the discrimination of small number sets only when they could use both number and continuous variables (including area of the figures), otherwise guppies failed the discrimination.

The contrasting findings we obtained for the experiments in which angelfish chose between shoals having large number of members or between shoals having small number of members support the hypothesis already suggested in previous studies [53], [55], [56]. Angelfish, similarly to other species, may be influenced by numerical and non-numerical features of the contrasted sets depending on the numerical size of these sets. Also this species may use different processing systems to discriminate small (signature limit 3–4) and large quantities (≥4). To control and systematically manipulate all possible continuous variables one may need to use computer animated images in a manner similar to the methods developed for zebrafish [68], [69]. This more rigorous control may also allow one to investigate whether the apparently distinct mechanisms utilized for small set comparisons vs. large sets comparisons are indeed distinct.
